# Comparative Analysis of Therapeutic Showers and Bathtubs for Pain Management and Labor Outcomes—A Retrospective Cohort Study

**DOI:** 10.3390/jcm13123517

**Published:** 2024-06-15

**Authors:** Elena Mellado-García, Lourdes Díaz-Rodríguez, Jonathan Cortés-Martín, Juan Carlos Sánchez-García, Beatriz Piqueras-Sola, Juan Carlos Higuero Macías, Francisco Rivas Ruiz, Raquel Rodríguez-Blanque

**Affiliations:** 1 Department of Nursing, Faculty of Health Sciences, University of Granada, 18016 Granada, Spain; e.elenamellado@go.ugr.es (E.M.-G.); cldiaz@ugr.es (L.D.-R.); jcortesmartin@ugr.es (J.C.-M.); rarobladoc@ugr.es (R.R.-B.); 2 Virgen de las Nieves University Hospital, 18014 Granada, Spain; beatriz.piqueras.sspa@juntadeandalucia.es; 3 Costa del Sol University Hospital, 29603 Marbella, Spain; juancarlos.higueromacas@gmail.com (J.C.H.M.); francisco.rivas.ruiz.sspa@juntadeandalucia.es (F.R.R.); 4 San Cecilio University Hospital, 18016 Granada, Spain

**Keywords:** hydrotherapy, waterbirth, immersion, first labor stage, maternal health

## Abstract

Hydrotherapy, including the use of therapeutic showers and bathtubs, has been studied for its potential benefits in labor pain management. Previous research has indicated that hydrotherapy can alleviate pain, but comparative studies between therapeutic showers and bathtubs are scarce. **Objective**: This study aims to compare the effects of therapeutic showers and bathtubs on pain perception, labor duration, use of epidural analgesia, and maternal and neonatal outcomes during labor. **Methods**: A total of 124 pregnant women were included in this study. Participants were divided into two groups: those who used a therapeutic shower and those who used a bathtub during labor. Pain levels were measured using a visual analog scale (VAS). Labor duration, use of epidural analgesia, types of delivery, maternal outcomes (postpartum hemorrhage, perineal status, maternal hypotension, fever, and breastfeeding), and neonatal outcomes (APGAR scores, fetal heart rate, complications, and neonatal unit admissions) were recorded and analyzed. **Results**: Both the therapeutic shower and the bathtub effectively reduced pain perception, with the bathtub showing a greater reduction in VAS scores. The therapeutic shower group experienced a significantly shorter labor duration compared to the bathtub group. The majority of participants in both groups did not require epidural analgesia, with no significant differences between the groups. There were no significant differences in the types of delivery. Maternal outcomes indicated a lower incidence of perineal tears and episiotomies in the therapeutic shower group. Neonatal outcomes, including APGAR scores and fetal heart rate, were similar between the groups, with no significant differences in complications or neonatal unit admissions. **Conclusions**: Both therapeutic showers and bathtubs are effective for pain relief during labor, with the bathtub showing a higher reduction in pain intensity. The therapeutic shower is associated with a shorter labor duration and a lower incidence of perineal tears and episiotomies. Both methods are safe for neonatal well-being, making hydrotherapy a viable non-pharmacological option for pain management in labor. However, the therapeutic shower may offer additional benefits in terms of labor duration and maternal outcomes.

## 1. Introduction

Hydrotherapy as a method for managing pain during childbirth has been used for thousands of years, and its exact origin is unknown [1]. Currently, many women seek non-pharmacological methods for pain relief during labor. The use of hydrotherapy can provide natural pain relief because warm water helps relax muscles and can reduce the sensation of pain, allowing women to better manage contractions [1,2].

The use of warm water can help reduce anxiety and stress, promoting a state of calm that facilitates the birthing process. The option to choose hydrotherapy for pain management during labor can give women a sense of control and empowerment over their birthing experience [3]. It allows them to actively participate in their care and make informed decisions about pain management. It is important to consider that each woman has her own preferences and needs during labor, and the decision to use hydrotherapy should be individualized [4].

In 2022, the Health Technology Assessment Service of the Basque Country (OSTEBA), supported by the Spanish Ministry of Health, published a comprehensive report on water immersion during labor. This study focused on two main aspects: evaluating the efficacy, effectiveness, and safety of water immersion during labor, and understanding the values and preferences of women who had experienced this birthing method. Given existing concerns, particularly regarding the safety of the newborn, the report aimed to analyze the available evidence to determine the safety and efficacy of water immersion during labor for both the mother and the neonate [5].

According to the literature review, various studies have been conducted to demonstrate the efficacy of therapeutic showers during labor for pain relief, compared to women who do not use water during the birthing process [6,7]. However, as of our review, we have not found specific studies that directly compare therapeutic showers and bathtubs in this context. Nevertheless, there are articles that compare therapeutic showers with other non-pharmacological methods, such as the use of perineal exercises with a Swiss ball during the dilation phase. These studies have yielded equally interesting results, demonstrating that the combination of therapeutic showers with these exercises is associated with reduced pain during labor and greater comfort for the mother [8].

In a pretest-posttest design study with a single group of 24 women who used the therapeutic shower for 30 min, numerical pain rating scales were evaluated before and after use. A significant decrease in both pain perception and levels of tension and anxiety was observed after the intervention [9].

Currently, in Spain, maternity units are incorporating bathtubs in their delivery rooms, but not all hospitals in the country can offer these services due to a lack of necessary infrastructure, specifically bathtubs for water immersion use by pregnant women during labor. Some delivery rooms have therapeutic showers available, but these do not provide full water immersion. The purpose of the present study was to compare whether therapeutic showers can be as effective as bathtubs regarding labor duration, use of analgesia, pain relief, and maternal and fetal outcomes.

### Objectives

To evaluate and compare the effects of using a bathtub and a therapeutic shower during labor on pain perception, the use of epidural analgesia, labor duration, and maternal and fetal outcomes.

## 2. Materials and Methods

### 2.1. Study Design

This is a retrospective cohort study of women who chose to use hydrotherapy during their labor. The report of this research follows the STROBE guidelines for observational studies. The study was conducted in accordance with the Declaration of Helsinki for research involving humans and was approved by the Ethics Committee of Hospital Costa del Sol (002_oct18_PI-hydrotherapy in labor) in November 2018.

### 2.2. Setting

These are secondary outcomes from a study that evaluated the use of hydrotherapy during labor. The initial study included women who gave birth at Hospital Costa del Sol, Málaga (Spain), during the period between January 2010, when hydrotherapy began to be offered during labor at the hospital, and December 2020. In this hospital, the use of hydrotherapy is indicated in the first stage of labor, either through a therapeutic shower or by immersion in a bathtub. Data were collected from each woman’s partogram as well as from the medical records of both the mother and the newborn.

### 2.3. Participants

Our study included women with low-risk pregnancies and labors, which means they had a healthy singleton pregnancy, a body mass index of 30 kg/m^2^ or less, cephalic presentation, spontaneous onset of labor, a gestational age between 37 + 0 and 41 + 6 weeks, and a normal cardiotocographic record upon admission. Women with multiple pregnancies and those who gave birth before 37 weeks or after 42 weeks were excluded. According to the protocol of our Labor Unit, all admitted women were offered the option to use hydrotherapy during the labor process. The participants in this study had no history of opioid medication use.

### 2.4. Variables and Data Sources

The study meticulously planned data coding in advance, extracting data directly from medical records into a structured database. It analyzes a variety of variables related to labor and hydrotherapy. Regarding pain relief during labor, pain perception was assessed using the visual analogue scale (VAS) in both the therapeutic shower group and the bathtub group, as well as comparing the median pain scores before and after the use of each method. Regarding labor duration, dilation times and overall labor time were examined in both groups. The use of epidural analgesia during labor was also recorded. In terms of delivery types, the proportion of spontaneous and operative deliveries in each group was observed. Additionally, various maternal outcomes were explored, including the incidence of postpartum hemorrhage, perineal status, presence of hypotension, maternal fever, and breastfeeding. As for neonatal outcomes, APGAR scores, fetal heart rate, fetal complications, and neonatal unit admission were analyzed.

### 2.5. Bias

To mitigate potential biases, the study established precise inclusion and exclusion criteria for participants, ensured data anonymity, and conducted meticulous data coding. Additionally, confounding variables were controlled through multivariable statistical analysis. These measures ensured the validity and reliability of the findings obtained in this retrospective cohort study.

### 2.6. Study Size

The sample size for this study was determined using the same parameters and methodology as the previously published initial study [10]. For the primary objective of comparing the duration of the first stage of labor between the hydrotherapy group and the non-hydrotherapy group, a statistically significant difference of 16 min between the groups was considered. Based on the study by Torkamani, Kangani, and Janani (2010) [11], a standard deviation of 48 min was used for each group. With a type I error (alpha) of 0.05 and a type II error (beta) of 0.20, it was determined that 111 patients per group were required. Considering a 10% loss rate in the evaluation of medical records, the total sample size was adjusted to 248 patients, evenly distributed as 124 patients per group.

To ensure the robustness of the results and to study additional data of interest, the sample was expanded to a total of 377 women, with 253 individuals in the control group and 124 in the hydrotherapy experimental group. This approach allowed us to further explore various relevant clinical and demographic aspects while maintaining consistency with the originally calculated sample size from the initial study.

### 2.7. Statistical Methods

Descriptive analysis was performed using measures of central tendency, dispersion, and position (median and interquartile range (P75–P25)) for quantitative variables and frequency distribution for qualitative variables. To assess differences between study groups (bath vs. shower), the chi-squared test (or Fisher’s exact test if expected frequencies were less than 5) was used for qualitative variables, while Student’s *t*-test (or Mann–Whitney U test if the distribution was non-normal) was used for quantitative variables. Using pain as the outcome variable, a multivariate linear regression model was employed, including unbalanced independent variables from previous bivariate analysis, selecting variables with a criterion of *p* < 0.05, and describing the Beta coefficient (β) with respective 95% confidence intervals (CI95%). This involved checking for normality, homoscedasticity, and multicollinearity.

For all analyses, the level of statistical significance was set at *p* < 0.05. The analysis was performed using SPSS vs. 28.0 program for Windows (IBM Corporation, Armonk, NY, USA) statistical software.

### 2.8. Ethics Statement

This study was conducted in accordance with the Declaration of Helsinki for research involving human subjects. The Ethics Committee of the Costa del Sol Hospital approved the study in November 2018 under reference number 002_oct18_PI-hydrotherapy birth, ensuring the ethical compliance of the research.

No personal or identifying information was collected. Anonymity was guaranteed by the research service of Hospital Costa del Sol, which anonymized the personal or identifying data of the women involved in the study. Additionally, the data were stored on a password-protected personal computer.

## 3. Results

The results examined the effect of hydrotherapy, both in the form of a bathtub and a therapeutic shower, in relation to pain relief during labor, its duration, the use of pharmacological analgesia, and delivery types. Additionally, maternal and fetal outcomes were analyzed based on whether water immersion in a bathtub or the use of water in a therapeutic shower was performed.

**Figure 1:**
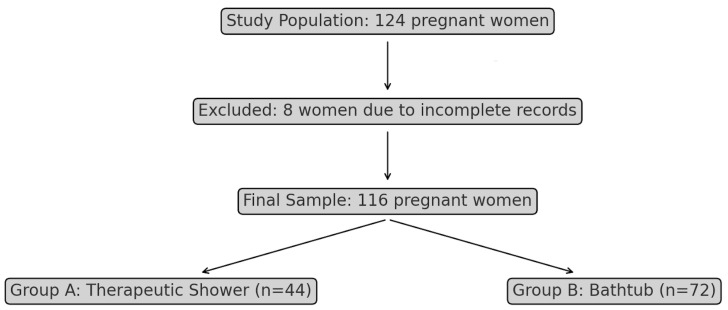
Flow diagram.

For this study, a sample of 124 laboring women was recruited using a systematic sampling approach. This included 44 women (35.5%) who utilized the therapeutic shower and 80 women (64.5%) who immersed themselves in a bathtub with water immersion (Figure 1).

To determine if there were significant differences between the groups, the obstetric characteristics of the sample were evaluated, which are presented in Table 1:

These results demonstrate the distribution of key obstetric characteristics between women who used a therapeutic shower and those who used a bathtub during labor. No statistically significant differences were found between the groups in terms of age, grouped gestation, history of abortions, or the number of previous children.

As shown in Table 2, the primary and secondary outcomes of our study indicate significant differences in the total labor time and intact perineal state between the groups using the therapeutic shower and the bathtub. However, no significant differences were found in the use of epidural analgesia, types of delivery, or the incidence of maternal fever and breastfeeding.

### 3.1. Pain Relief

The initial findings of this study revealed statistically significant differences between the use of hydrotherapy during labor compared to non-use, regardless of whether a therapeutic shower or bathtub was utilized during labor [10]. At this juncture, we scrutinized the sensation of pain in the bathtub versus the therapeutic shower.

Our sample, comprised of 124 pregnant women, furnishes comparative data on perceived pain during the use of therapeutic showers and bathtubs. In the therapeutic shower group, eight cases were lost, while in the bathtub group, five cases were lost due to lack of recording.

**Figure 2:**
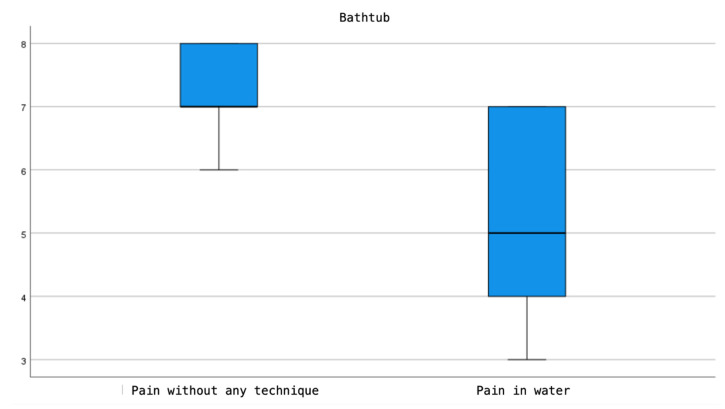
Comparison of Pain Intensity in the Bathtub, Before and After Use.

**Figure 3:**
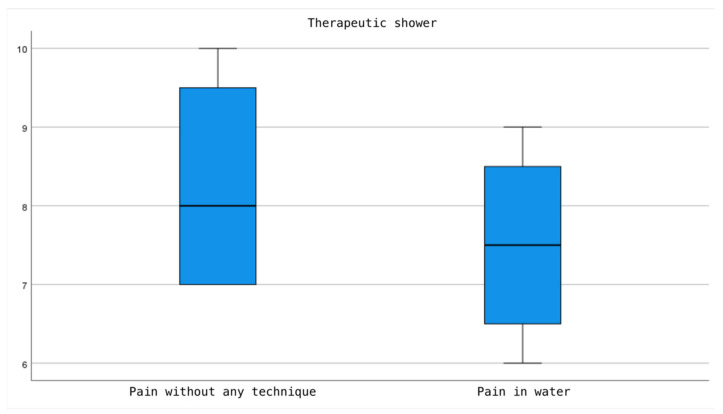
Comparison of Pain Intensity in the Therapeutic Shower, Before and After Use.

According to the results presented, both the use of therapeutic showers and bathtubs show a reduction in pain perception compared to the sensation of pain prior to their use. However, this decrease is more pronounced in the bathtub group, with the difference in the pain perception scale before and after use being statistically significant (*p* = 0.003). In contrast, in the therapeutic shower group, although there is a noticeable reduction in pain perception, this difference does not reach statistical significance (*p* = 0.083) (Figure 2 and Figure 3).

### 3.2. Duration of Labor

Upon examining the results between the group using the therapeutic shower and the group using the bathtub, it was found that dilation times and overall labor duration showed significant differences between the two groups, favoring the group that used the therapeutic shower (Table 3).

### 3.3. Use of Analgesia

A total of 99 pregnant women did not use epidural analgesia, representing 79.8% of the 124 women in our study. The comparison between groups yielded a non-significant result, indicating no association between epidural use and the bathing method, whether therapeutic shower or bathtub.

### 3.4. Types of Delivery

The data comparing the therapeutic shower group and the bathtub group, as well as the types of delivery, are very similar. According to the *p*-values obtained, none of the statistical tests performed indicate a statistically significant association between the type of delivery and the bathing method. All *p*-values are well above the 0.05 threshold. Therefore, no significant differences were found between the use of the bathtub and the therapeutic shower concerning the types of delivery. The analysis indicates that in the therapeutic shower group, 2.3% of deliveries were operative vaginal and operative cesarean, while 97.7% were spontaneous vaginal. In the bathtub group, 2.5% of deliveries were operative vaginal and operative cesarean, and 97.5% were spontaneous vaginal.

### 3.5. Maternal Outcomes

The effect of the therapeutic shower and bathtub on various maternal parameters has been investigated:

#### 3.5.1. Postpartum Hemorrhage

There were two cases of postpartum hemorrhage in the therapeutic shower group and four cases in the bathtub group, representing 4.5% and 5.0% of the sample, respectively. However, no statistically significant differences were found regarding this variable.

#### 3.5.2. Postpartum Perineal Status 

The study results indicate a statistically significant decrease in the frequency of 1st, 2nd, and 3rd-degree tears, as well as episiotomies, in favor of the group that used the therapeutic shower. In the therapeutic shower group, 45.5% of women had an intact perineum after delivery, compared to 23.8% in the bathtub group. The incidence of 1st, 2nd, and 3rd-degree tears and episiotomies was 54.5% and 76.3%, respectively. The *p*-value of 0.022 suggests that the use of the therapeutic shower was associated with a lower incidence of tears and episiotomies compared to the use of the bathtub.

#### 3.5.3. Maternal Hypotension 

It was observed that 9.1% of women in the therapeutic shower group experienced hypotension, compared to 3.8% of women who used the bathtub. However, the *p*-value of 0.244 does not show statistically significant differences between the groups.

#### 3.5.4. Maternal Fever 

In the therapeutic shower group, no cases of fever were recorded, while in the bathtub group, there was one case with a fever above 38 °C. No significant differences were found between the groups concerning this variable.

#### 3.5.5. Breastfeeding 

No statistically significant differences were recorded (*p* = 1.000); both percentages were high, with 95.5% for women who used the therapeutic shower compared to 96.3% for those who used the bathtub.

### 3.6. Neonatal Outcomes

Regarding fetal parameters, the analysis between the groups revealed no significant differences in APGAR scores at 1 and 5 min, except for one case in the bathtub group with an APGAR score at 1 min below 7. Fetal heart rate (FHR) was normal in 94.4% of cases in both groups. Specifically, in the therapeutic shower group, 6.8% had a non-reassuring fetal cardiotocographic record (FCTG), while in the bathtub group, this percentage was 5%. No significant differences were found in the APGAR and FCTG variables.

Regarding fetal complications and neonatal unit admissions (NICU), 119 newborns did not have complications, and 118 did not require NICU admission, representing 96% and 95.2% of the sample, respectively. Fetal complications occurred in 5% of the newborns in the bathtub group and 2.3% of the newborns in the therapeutic shower group. NICU admission occurred in 5% of the newborns in the bathtub group and 4.5% of the newborns in the therapeutic shower group. No significant differences were found for these two variables, thus no relationship could be established between the method of water use during labor and the presence of fetal complications or NICU admissions.

## 4. Discussion

We focused on investigating the effect of hydrotherapy during labor, according to the use of a bathtub or therapeutic shower, in relation to perceived pain, labor duration, analgesia use, and maternal and neonatal outcomes. The objective is to contribute to the scientific evidence by comparing these two groups, which is uncommon due to the scarcity of literature addressing this comparison.

Pain management is a fundamental aspect of labor care, which is why it has been the subject of numerous scientific investigations studying its relationship with non-pharmacological methods such as hydrotherapy. Publications analyzing both the therapeutic shower and the bathtub encompassed in hydrotherapy in general emphasize how the sensation of pain can decrease through the use of hydrotherapy. Our study also corroborates these findings: the comparison between the groups shows that the bathtub reduces the sensation of pain by one point more on the visual analog scale (VAS) compared to the therapeutic shower. Other studies, such as the one conducted by Davim et al. [12], have observed that pain relief increases as dilation progresses during labor when using the therapeutic shower. In a clinical trial conducted by Lee et al. [6], it was demonstrated that the therapeutic shower is a cost-effective, comfortable, and easy-to-perform non-pharmacological method for reducing pain, with positive results on a visual analog pain scale. A systematic review by Vargens, Silva, and Progianti [3] compiled 21 articles on the use of hydrotherapy and concluded that both the bathtub and the therapeutic shower effectively reduce pain during labor.

Our study also shows that the therapeutic shower results in a shorter labor duration compared to the use of the bathtub. Numerous studies discuss the use of the bathtub as a pain relief method [13,14,15,16,17], while there are also studies addressing the use of the therapeutic shower [6,7,12,13,18]. A decrease in dilation time and total labor duration has been observed when using the therapeutic shower as a method. Gallo et al. [13] detailed in their randomized trial how a warm shower at more than 7 cm dilation, combined with exercises on a Swiss ball and lumbosacral massage before 7 cm, yielded significant benefits, such as a reduction of 72 min compared to the group that did not use non-pharmacological techniques during labor, as well as differences in faster expulsion times.

Regarding specific research on analgesia use, the systematic review by Cluett et al. [1] revealed discrepancies in the use of epidural analgesia among women who opted for water immersion during the first stage of labor and those who did not. It was observed that in the group of women who experienced water labor, a smaller proportion opted for epidural analgesia compared to the groups that did not use water as a pain relief method. However, no significant differences were found in the use of epidural analgesia or the use of pethidine/narcotics between the different groups. In our study, we found a significant association between the use of epidural and the use of hydrotherapy, either in a bathtub or therapeutic shower, considering that the majority of pregnant women who used the therapeutic shower or bathtub did not use epidural analgesia. Authors like Gallo et al. [13] and Stark [7] describe the therapeutic shower as one of the beneficial non-pharmacological interventions, with few side effects or contraindications, allowing for a reduction in pain perception and even reducing the use of epidural analgesia, although Stark’s study [7] found similar use of epidural analgesia in both the therapeutic shower group and the control group.

The randomized trial by Gallo et al. [13] not only studied variables such as pain and labor duration in women who used the therapeutic shower but also examined other parameters similar to those measured in our study. However, it is important to note that Gallo et al.‘s study compared the use of the therapeutic shower with exercises on a Swiss ball and lumbosacral massage. Among the results, neonatal effects stood out: the experimental group had a lower risk of respiratory distress and significantly better Apgar scores. However, no significant differences were observed regarding delivery types, perineal status, or obstetric complications. In our study, we also evaluated these parameters and found no significant differences, except in postpartum perineal status, where we observed a decrease in the frequency of tears and episiotomies in the group that used the therapeutic shower.

The main limitation was the lack of exhaustive records in medical histories, leading to a sample of sixteen pregnant women, as previous information was not typically recorded in these histories. Another limitation was the absence of data related to the water temperature of the bathtub or therapeutic shower, information that would have been useful to assess its possible impact on the health of pregnant women and fetal development. Water temperature could influence various physiological factors, such as blood circulation and muscle relaxation, in addition to preventing risks associated with extreme temperatures, such as overheating or thermal shock. Additionally, the retrospective nature of the study conducted at a single institution is a significant limitation, predisposing the results to considerable bias. This characteristic prevents the generalization of the findings to other populations or contexts.

## 5. Conclusions

The study demonstrates that hydrotherapy, through the use of both bathtubs and therapeutic showers, effectively reduces pain perception during labor. The bathtub, in particular, provides a slightly higher pain relief compared to the therapeutic shower. Moreover, the therapeutic shower is associated with a shorter labor duration. Despite these benefits, it is important to acknowledge the limitations, such as the retrospective nature of the study conducted at a single institution, which may introduce significant bias and limit the generalizability of the results. Further research with larger, multicenter studies is needed to validate these findings.

## Figures and Tables

**Table 1:** Baseline characteristics of the sample.

Variables		Therapeutic Shower	Bathtub	Total	*p*-Value
Age; Mean ± SD		30.70 ± 5.083	32.25 ± 5.784		0.140
Gestation grouped; *n* (%)	1	19 (43.2%)	43 (53.8%)	62 (50%)	0.549
	2	17 (38.6%)	21 (26.3%)	38 (30.6%)	
	3 or more	8 (18.2%)	16 (20%)	24 (19.4%)	
Abortions; *n* (%)	Absence	36 (81.8%)	59 (73.8%)	95 (76.6%)	0.427
	Presence	8 (18.2%)	21 (26.3%)	29 (23.4%)	
Previous Children	0	23 (52.3%)	54 (67.5%)	77 (62.1%)	0.139
	1 or more	21 (47.7%)	26 (32.5%)	47 (37.9%)	

**Table 2:** Summary of Primary and Secondary Outcomes.

Outcomes	Therapeutic Shower Group	Bathtub Group	*p*-Value
Primary Outcomes			
Total labor time (minutes)	155 (96.25–242.5)	227.5 (141.25–403.75)	0.004
Use of epidural analgesia	7 (15.9%)	18 (22.5%)	0.521
Types of delivery (% spontaneous)	97.7%	97.5%	>0.05
Pain perception before (VAS)	8 (7–9)	7 (7–8)	-
Pain perception after (VAS)	7.5 (6.25–8.75)	5 (4–7)	-
Secondary Outcomes			
Postpartum hemorrhage	2 (4.5%)	4 (5.0%)	>0.05
Intact perineal state	45.5%	23.8%	0.022
Maternal hypotension	9.1%	3.8%	0.244
Maternal fever	0%	1 (1.3%)	>0.05
Breastfeeding	95.5%	96.3%	1.000
APGAR score at 1 min (median, IQR)	Not specified	Not specified	Not specified
APGAR score at 5 min (median, IQR)	Not specified	Not specified	Not specified
Fetal heart rate	No specified complications	No specified complications	>0.05

**Table 3:** Results of Labor Duration by Stages in the Use of Bathtub and Therapeutic Shower.

Therapeutic Shower vs. Bathtub			Dilation Time	Expulsive Time	Placental Expulsion Time	Total Labor Time
Therapeutic shower	*n*	Valid	44	44	44	44
		Missing	0	0	0	0
	Median		90	31	10	155
	Percentile	25	56.25	16.25	10	96.25
		75	133.75	53.75	16.75	242.5
Bathtub	*n*	Valid	80	80	80	80
		Missing	0	0	0	0
	Median		150	44	10	227.5
	Percentile	25	93.75	15	10	141.25
		75	240	90	15	403.75
*p* valor			0.002	0.167	0.865	0.004

## Data Availability

Data regarding this study are available upon request from the corresponding author.
